# The chemokine landscape: one system multiple shades

**DOI:** 10.3389/fimmu.2023.1176619

**Published:** 2023-05-11

**Authors:** Valentina Cecchinato, Veronica Martini, Edisa Pirani, Elaheh Ghovehoud, Mariagrazia Uguccioni

**Affiliations:** Institute for Research in Biomedicine, Università della Svizzera italiana, Bellinzona, Switzerland

**Keywords:** Chemokines, Antagonism, Repulsion, Synergism, CXCL12/HMGB1 heterocomplex, Autoantibodies

## Abstract

Leukocyte trafficking is mainly governed by chemokines, chemotactic cytokines, which can be concomitantly produced in tissues during homeostatic conditions or inflammation. After the discovery and characterization of the individual chemokines, we and others have shown that they present additional properties. The first discoveries demonstrated that some chemokines act as natural antagonists on chemokine receptors, and prevent infiltration of leukocyte subsets in tissues. Later on it was shown that they can exert a repulsive effect on selective cell types, or synergize with other chemokines and inflammatory mediators to enhance chemokine receptors activities. The relevance of the fine-tuning modulation has been demonstrated *in vivo* in a multitude of processes, spanning from chronic inflammation to tissue regeneration, while its role in the tumor microenvironment needs further investigation. Moreover, naturally occurring autoantibodies targeting chemokines were found in tumors and autoimmune diseases. More recently in SARS-CoV-2 infection, the presence of several autoantibodies neutralizing chemokine activities distinguished disease severity, and they were shown to be beneficial, protecting from long-term sequelae. Here, we review the additional properties of chemokines that influence cell recruitment and activities. We believe these features need to be taken into account when designing novel therapeutic strategies targeting immunological disorders.

## Introduction

Chemokines, chemotactic cytokines, engage in a promiscuous fashion a panel of over 20 chemokine receptors, key regulators of leukocyte migrations and functions. The chemokine system includes approximately 50 ligands, which play a fundamental role both in physiological and pathological immune responses (1).

The three-dimensional structures of all chemokines, as determined by nuclear magnetic resonance (NMR) spectroscopy or by X-ray crystallography, reveal remarkably similar protein backbones, tied together by two disulfide bonds formed among the four cysteines conserved in almost all chemokines. In all known structures, the N-terminal domain is unordered and contains two of the four cysteines. The loop region after the second cysteine, often referred to as “the N-loop”, is followed by three antiparallel β−strands and a C-terminal α−helix, all of which are connected by short, random-coiled loops. The proximity of the cysteines in the N-loop has been used to classify chemokines in subfamilies: CC, where the cysteines are adjacent, CXC, in which one amino acid is interposed between the cysteines and CX_3_C, where three amino acids are present in between, while XC chemokines lack the first and the third conserved cysteine (2–4).

To mediate their activity, chemokines bind to cell surface receptors, which belong to the largest branch of the γ subfamily of rhodopsin-like G protein-coupled receptors (GPCRs). Today, 19 signaling receptors have been identified: 6 CXCRs, (CXCR1-6), 10 CCRs (CCR1-10), CX3CR1 and XCR1 (5). In addition, there are four “atypical” receptors (ACKR1-4) that use β-arrestins to elicit their functions. Atypical chemokine receptors impacts chemokine availability by scavenging and degrading the chemokine in the lysosomes, or by transporting the chemokines across different barriers *via* transcytosis (6).

Initial studies on structure-function relationships of chemokines were performed with CXCL8, using N-terminal truncations and amino acid substitutions. NMR−studies of CXCL8 in complex with peptides derived from the N-terminus of CXCR1, together with single-site mutagenesis, led to the identification of the major receptor-binding region: a positively charged groove between the N-loop and the third β−strand, into which the N−terminus of the receptor binds (7). Further studies of other chemokine receptor-peptide complexes corroborated this model (2).

Despite the apparent redundancy within the chemokine system, characterized by multiple chemokines binding to a single receptor and one receptor being activated by multiple chemokines, the system has been demonstrated to exhibit a high degree of specificity and complexity. Chemokine receptors are selectively expressed on specific subsets of cells, which contributes to their functional characteristics and homing abilities. Their ligands, on the other hand, can be expressed either individually or in combination within a particular tissue, both under normal and pathological conditions (8).

Studies investigating expression of chemokines in human samples from different diseases have revealed that many chemokines can be produced during the disease process (9). *In vivo* models and *in vitro* studies have highlighted the importance of chemokine binding to extracellular matrix components, and their activities as complexes (10). However, in pathological conditions, chemokine production does not always entirely account for the disease characteristics. This discrepancy might be partially explained by the additional chemokine activities and their natural regulation that we, and others, have described in the last three decades (**Table 1**). This review aims to summarize these findings and their relevance to disease progress and treatment.

**Table 1:** Modulators of chemokine activities.

Activity	Receptor	Modulator	Reference
Antagonism	CCR1	CCL4 CCL26	(11) (12)
	CCR2	CCL2 (9–76) CCL11	(13) (14)
	CCR3	CCL18 CXCL9 CXCL10 CXCL11	(15–17)
	CCR5	CCL7 CCL26 CXCL11	(18) (12) (19)
Repulsion	CCR2	CCL26	(20, 21)
	CXCR4	CXCL12	(22–24)
Synergism	CCR2	CCL19 CCL21	(25)
	CCR4	CXCL10	(26)
	CCR5	CXCL4	(27)
	CCR7	CXCL13	(28)
	CXCR3	CXCL12	(29, 30)
	CXCR4	CXCL9 HNP1 Galectins HMGB1	(31) (32) (33) (34, 35)

## Natural chemokine antagonists

The concomitant expression of several chemokines in inflamed tissues led us to explore the possibility that leukocyte infiltration may occur stepwise in response to gradients of different chemokines, and that chemokines can modulate, as natural antagonists, the activity of receptors that are different from their “traditional” target ones.

Chemokine receptor antagonism by unmodified naturally occurring ligands, therefore, constitutes a potentially important regulatory principle of chemokine-driven reactions.

**Figure 1:**
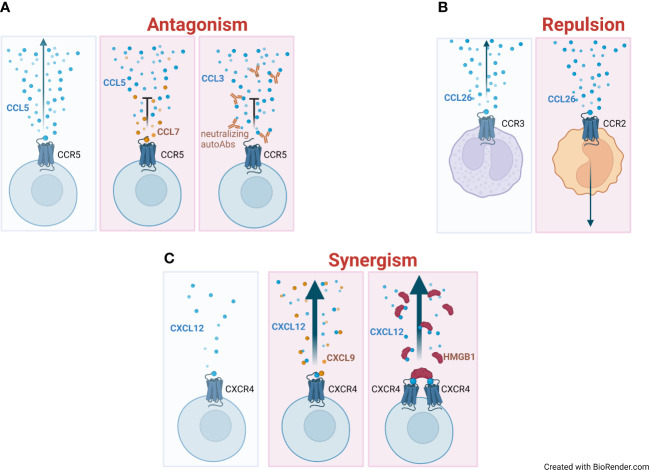
Mechanisms of regulation of chemokine activities. **(A)** Directional migration (indicated with an arrow) of a chemokine (blue circles) on its respective receptor, and the gradient formation (left panel). Antagonistic effect and impairment of migration elicited by the co-presence of high levels of a non-agonist chemokine (CCL7, orange circles) (middle panel) or by neutralizing autoantibodies (autoAbs) targeting the chemokine (right panel). **(B)** Repulsive effect of CCL26 on monocytes that move away from the chemokine gradient (right panel) compared to eosinophils, which are attracted by it (left panel). **(C)** Absence of migration at low concentrations of the agonist (blue circles) (left panel). Enhanced migration (represented as a thick arrow) in the presence of an heterocomplex formed at low concentration of the agonist (CXCL12) and concomitant high concentration of a non-agonist chemokine (CXCL9, orange circles) (middle panel) or the alarmin High Mobility Group Box 1 (HMGB1) (red half-circles) (right panel).

Several reports have described that natural chemokine antagonists block the migration of inflammatory cells or provide an additional mechanism for selecting the leukocyte sub-type to be recruited at site of inflammation (**Figure 1A**).

A thorough pharmacological characterization of CCR5 revealed CCL7, a promiscuous agonist for CCR1, CCR2, and CCR3, as a highly potent, complete antagonist (18). An extensive study by Loetscher et al. showed CXCL11, an agonist of CXCR3, to be a highly selective and potent antagonist for CCR3, while CXCL9 and CXCL10, the other two agonists of CXCR3, were less efficient inhibitors (15). CCL18 and CXCL11 have independently been confirmed as CCR3 antagonists (16, 17). We have reported on the antagonistic effects of CCL11 on CCR2 (14), while another report characterized CCL11 as a partial antagonist of CCR2 (36). Similarly, CCL4 has been described as an antagonist (11) or partial agonist (37) for CCR1, possibly depending on the cellular background. Furthermore, we have characterized CCL26 as a natural antagonist for CCR1 and CCR5 (12), and CXCL11 for CCR5 (19).

In addition, naturally occurring post-translational modifications of chemokines, such as proteolytic processing of the N-terminus domain (38), as well as synthetic N−terminal truncated forms (13) have been shown to have antagonist activity. As an example, a truncated variant of CCL2 (MCP- 1 (9–76)) prevents the onset of arthritis, and reduces symptoms and cellular infiltrates in the MRL-*lpr* mouse model. Despite this important finding, clinical trials aimed at blocking CCL2/CCR2 interaction in Rheumatoid Arthritis (RA) failed to reach phase III (5), most likely due to the complexity of the disease in humans (*e.g.*: synovial infiltrate with predominant follicle like structure, monocyte/macrophages, or fibroblasts) and the different chemokines produced.

The unmodified natural antagonists act similarly to the truncated variants, which lack the N-terminal motif, and bind to the receptors *via* the structurally conserved N−loop and third β−strand, but then present the receptor with an N-terminal motif that is incapable of activating it. This notion is supported by experiments showing that a CCL11 variant, featuring the N−terminal motif of CXCL11 instead of its native one, acted as a highly potent CCR3 antagonist (39).

## Repulsive chemokines

A second feature of chemokines is their potential to exert a repulsive effect on selected cell types (**Figure 1B**). This feature was first described in 2000 by Poznansky and colleagues: high concentrations of CXCL12 exert a repulsive rather than an attractive effect on mature T cells (22).

While low concentrations of the chemokine attract a variety of leukocyte subpopulations, high concentrations of CXCL12 induce a repulsive effect on both naïve and memory CD4^+^ and CD8^+^ T cells, which move away from the source of the stimulus. The repulsive effect of CXCL12 on T cells is mediated by distinct signaling pathways as compared to those involved in chemotaxis: while the first is inhibited by cAMP agonists, the second requires tyrosine kinase activity, and they both depend on CXCR4, Gαi protein and phosphatidylinositol 3-kinase activities. Differences in i) receptor dimerization and internalization, ii) signal transduction pathways elicited by surface or endocytosed receptor/ligand complexes, and iii) ratio between GAG-bound and free CXCL12, have been theorized as mechanisms involved in the different activity exerted by low or high chemokine concentrations, but no definitive experimental evidence has been provided to support these possibilities (40). It has been speculated that the repulsive effect exerted by CXCL12 on T cells prevents their infiltration in organs that produce abundant amounts of the chemokine, such as the bone marrow or the thymus, or can act as a limiting mechanism to avoid excessive accumulation of lymphocytes at site of inflammation to avoid self-perpetuating immune responses.

The relevance of the repulsive effect of CXCL12 on T cells in physiology has been later proven in the setting of mature thymocytes emigration from the thymus (23). Mature single positive CD4^+^ and CD8^+^ thymocytes, but not immature triple negative or double positive thymocytes, migrate away from thymic fragments through a *Bordetella Pertussis* toxin sensitive process, indicating that Gαi protein-coupled receptors are responsible for the active movement of cells away from the tissue. Lack of a negative CXCL12 gradient and/or inhibition of CXCR4 prevent thymocytes emigration from thymic fragments, thus demonstrating that the repulsive effect is dependent on the presence of CXCR4 on mature thymocytes and on the high concentrations of CXCL12 produced by the thymic stroma.

Elevated levels of CXCL12 are also found in dysplastic tissues, such as primary brain tumors, melanomas, and ovarian carcinomas. Despite high concentrations of the chemokine, these tumors are rarely infiltrated by T cells. In 2006, Vianello and colleagues provided evidence that melanomas expressing elevated levels of CXCL12 can repel T cells, thereby abrogating Antigen (Ag)-specific T cell infiltration into the tumor and allowing it to escape immune control (24). Melanomas engineered to express low or high concentrations of CXCL12 display distinct levels of tumor infiltrating lymphocytes. Ag-specific T cells infiltrate tumors expressing low levels of CXCL12, but not those expressing elevated levels of the chemokine. The infiltration of Ag-specific T cells into these tumors is therefore affected by the concentration of CXCL12, with low concentrations leading to chemotactic effects and high concentrations to repulsion. These activities are mediated by CXCR4, as Ag-specific T cell pre-treatment with the antagonist AMD3100 results in decreased infiltration of low CXCL12 expressing tumors, but abrogates the repulsive effect, and restores infiltration in tumors expressing high levels of CXCL12, possibly *via* different chemokine receptors.

The ability to induce migration away from the stimulus is not an exclusive feature of CXCL12. Indeed, we showed that CCL26 actively repulses monocytes (20), a finding that was later confirmed in an independent study (21). While both the chemotactic and repulsive effects exerted by different CXCL12 concentrations are mediated by CXCR4, the repulsive effect of CCL26 does not require the expression of its cognate receptor CCR3. This repulsive effect on monocytes depends on CCR2 availability, and requires Gαi protein and tyrosine kinase activities. Moreover, monocyte migration along a CCL2 gradient is significantly increased in the presence of an opposite CCL26 gradient, indicating that the concomitant presence of two opposite gradients in the microenvironment could enhance monocyte responses to CCL2. This effect could be relevant *in vivo* to direct monocytes from the blood stream to the site of inflammation, with CCL26 being expressed by vascular endothelial cells, during allergic reactions (41), and CCL2 by the inflamed tissue.

## Synergy-inducing chemokines

A third level of modulation in chemokine activity is their ability to interact with other chemokines or molecules in the microenvironment, leading to synergistic effects on leukocyte functions in response to chemoattractants (**Figure 1C**). This can occur through direct interactions between molecules or through engagement of different receptors on the same cell. The first description of this mechanism dates to 2002 when the bovine chemokine regakine−1 was discovered to induce an enhanced neutrophil migration when combined with CXCL7, CXCL8 and C5a (42). The receptor or the mechanism of regakine-1 induced synergism were not identified. Competition with labelled C5a for binding to neutrophils or receptor transfected cell lines demonstrated that regakine−1 does not alter receptor recognition. The protein kinase inhibitors 2’−amino−3’−methoxyflavone (PD98059), wortmannin and staurosporin had no effect on the synergy between C5a and regakine−1. This first observation was followed by the description that migration of natural type I IFN−producing cells, a subpopulation of murine and human lymphocytes, to the CXCR3 agonists requires stimulation of CXCR4 by CXCL12 (29). The mechanism by which CXCL12 induces enhanced migration in response to CXCR3 agonists remains unknown. CXCL12 does not upregulate the expression of CXCR3 and does not increase the affinity of CXCR3 for its agonists. Apart from chemotaxis, the authors did not investigate other cell functions, and did not assess the signaling pathways involved. Similarly, the same enhanced migration in response to CXCR3 agonists, induced by stimulation with CXCL12, was observed by Vanbervliet et al. on human plasmacytoid dendritic cells (30). These reports undoubtedly indicated, as for natural antagonist chemokines, the necessity to further investigate the mechanisms governing concomitant expression of chemokines and cell functions, fostering research in this area.

Assessing the activity of chemokine heterocomplexes, von Hundelshausen et al. demonstrated that the complex between two chemokines, CCL5 and CXCL4, triggers the arrest of monocytes on activated endothelium (27), while concomitantly we demonstrated that the heterocomplex between CXCL13 and CCL21 or CCL19 modulates CCR7-expressing cell activities (28). Likewise, CCR4^+^ lymphocytes migrate toward low concentrations of CCL22 in the presence of CXCL10 (26), monocytes migrate toward low concentrations of CCL7 in the presence of CCL19 or CCL21 (25), and lymphoma cells respond to the heterocomplex formed by CXCL12 and CXCL9 *via* CXCR4 (31).

Von Hundelshausen et al. extensively studied how human chemokines interact with each other and demonstrated their relevance in enhancing receptor triggering or inhibiting receptor activities (43). Several studied performed *in vivo* highlighted the importance of disrupting the heterocomplex formation to ameliorate inflammation (44–46).

Recently, a non-dissociating CXCL4–CXCL12 heterodimer was used as a new tool to further study chemokine-chemokine and chemokine heterodimer-receptor interactions in breast cancer cells (47).

Chemokine heterocomplexes might provide an amplification system, when the concentration of the agonist is too low to trigger a proper receptor response (48). On the other hand, when synergy-inducing molecules are present in an environment rich of the agonist, they could possibly dampen cellular responses, as indicated by *in vitro* migration (26, 28, 34).

Chemokine activity can also be modulated by their interaction with other molecules such as the alarmin High Mobility Group Box 1 (HMGB1) (34), as described in the next section, the α-defensin HNP1 (32), or galectins (33), further supporting the relevance of the tissue microenvironment in modulating cellular recruitment and responses in inflammation.

### CXCL12/HMGB1 heterocomplex

Damage-associated molecular patterns, also known as alarmins, are danger signals released upon tissue damage to activate the inflammatory response. The alarmin HMGB1 has been shown to form a heterocomplex with CXCL12, favoring cell migration *via* CXCR4 both *in vitro* and *in vivo*. This synergistic activity, demonstrated *in vitro* using mouse fibroblasts and monocytes, was further assessed in an *in vivo* model of sterile inflammation. Moreover, this effect is blocked by glycyrrhizin, which prevents the formation of the heterocomplex, by anti-CXCL12 antibodies, or by AMD3100, inhibiting CXCR4 activation (34).

**Figure 2:**
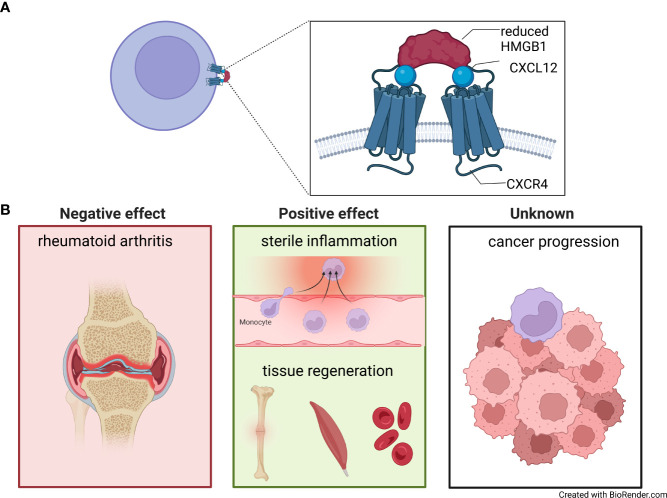
The CXCL12/HMGB1 heterocomplex. **(A)** Model of two molecules of CXCL12 (blue circle) complexed with reduced High Mobility Group Box 1 (HMGB1) (red half-circles), binding and triggering CXCR4 homodimers on the cell surfaces. **(B)** CXCL12/HMGB1 heterocomplex is present in rheumatoid arthritis, where it sustains inflammation in the synovial membrane (left panel). A positive effect of the CXCL12/HMGB1 heterocomplex has been shown in models of sterile inflammation, fracture healing, muscle repair and hematopoiesis after chemotherapeutic myeloablation (middle panel). The role of the heterocomplex in cancer has yet to be fully understood (right panel).

HMGB1 is characterized by two DNA binding domains, Box A and Box B, connected by a flexible linker region, and a long acidic C-terminal tail. In the tissue microenvironment, HMGB1 can be present in two different redox isoforms: reduced-HMGB1 in which the two conserved cysteines at position 23 and 45 within Box A are reduced, and oxidized-HMGB1 in which these two cysteines form a disulfide bond. Only reduced-HMGB1 synergises on CXCL12 activities (49). Results from NMR and Surface Plasmon Resonance experiments revealed that CXCL12 interacts separately with each of the HMGB1 boxes (34). Molecular dynamics simulations and protein-protein docking calculations showed that reduced-HMGB1 can accommodate two CXCL12 molecules, while oxidized-HMGB1 tends to be more compact and displays a lower accessible surface for the chemokine. Furthermore, only when the two CXCL12 bind to the reduced-HMGB1, their N-terminal domains are oriented in the best conformation to trigger CXCR4 dimers (**Figure 2A**) (50). Fluorescence Resonance Energy Transfer studies investigating CXCR4 dimer formation showed that the CXCL12/HMGB1 heterocomplex induces different rearrangements of CXCR4 homodimers on the cell surface, compared to CXCL12 alone, without altering the overall number of CXCR4 homodimers formed (34). Triggering of CXCR4 by the heterocomplex, in comparison to CXCL12 alone, results in a distinctive engagement of the β-arrestin proteins: β-arrestin1 for actin polymerization and β-arrestin2 for directional migration. In addition, CXCR4 is preserved on the cell surface and this results in an enhanced response to the chemotactic signal (51).

### CXCL12/HMGB1 heterocomplex in inflammation

The early recruitment of mononuclear cells at the site of inflammation, also driven by the CXCL12/HMGB1 heterocomplex, is an essential step to mount a proper immune response. In such cases, it is essential that HMGB1 is maintained reduced in the microenvironment.

The heterocomplex mediated migration was first assessed in an *in vivo* model of sterile inflammation, achieved by the injection of cardiotoxin into the muscle. Endogenous production of HMGB1, and subsequent heterocomplex formation with the CXCL12 expressed on endothelial cells, promotes a fast recruitment of CD11b^+^ mononuclear cells (34). In this model, pre-treatment with glycyrrhizin, completely abolishes leukocyte infiltration.

More recently, Ferrara and colleagues, monitored HMGB1 redox status and leukocyte infiltration in tissues, demonstrating that in early inflammation HMGB1 is mainly reduced, while oxidized-HMGB1 is prevalent at later stages, possibly due to the production of Reactive Oxygen Species by infiltrating leukocytes (52).

Despite its beneficial role in early leukocyte recruitment during acute inflammation, persistent expression of the heterocomplex might be detrimental and delay resolution of inflammation.

In patients with RA, an autoimmune condition affecting the joints, monocytes are known to be recruited *via* CXCL12 (53), and HMGB1 is found overexpressed in the synovial fluid (54), where the heterocomplex can be detected (35). Monocytes from RA patients with active disease migrate towards the CXCL12/HMGB1 heterocomplex formed at lower HMGB1 concentration, compared to monocytes obtained from healthy individuals, due to the production of thioredoxin and thioredoxin reductase, enzymes implicated in maintaining HMGB1 in its reduced form (35). *In vitro* treatment of monocytes from healthy individuals with prostaglandin E2, found at high concentration in the sera of RA patients, recapitulates the response to the heterocomplex of monocytes from active RA. This effect is abolished by celecoxib, a COX2 inhibitor used in RA treatment, which restores migration patterns to normal levels. Together, this study demonstrated the pathological role of CXCL12/HMGB1 heterocomplex in the recruitment of monocytes during active disease status, where they promote tissue damage (**Figure 2B**).

Indirect evidence points to a role for the CXCL12/HMGB1 heterocomplex in autoimmune disorders affecting the eyes. In a model of autoimmune uveitis, adoptive transfer of uveitogenic Ag−specific T cells promotes the active secretion of HMGB1 by retinal cells, a process mediated by Fas-FasL interaction (55, 56). Yun and colleagues demonstrated that, early after adoptive transfer, retinal cells actively secrete HMGB1 and subsequently produce CXCL12 (57). In addition, CXCR4 inhibition significantly reduces ocular leukocyte infiltration and improves clinical score. Although this represents the first indication of HMGB1 and CXCL12 pathological expression in uveitis, redox status of HMGB1 and therefore heterocomplex formation remains to be proven. Local upregulation of HMGB1 has been shown in vitreous samples of diabetic patients with ongoing retinopathy, and in the retinas of diabetic rat and mice models (58–60). The presence of CXCL12/HMGB1 heterocomplex has been demonstrated after intravitreal HMGB1 injection in diabetic mice, which upregulates CXCL12 (61). Future studies should investigate heterocomplex mediated leukocytes recruitment in the retina and how best to pharmacologically impair this migration.

A potential pathological role of CXCL12/HMGB1 has also been suggested for infectious diseases.

HMGB1 is secreted locally in response to *Pseudomonas aeruginosa* lung infection (62). De Leo and colleagues have recently demonstrated, in an *in vivo* model, that a newly discovered CXCL12/HMGB1 heterocomplex inhibitor, pamoic acid, can reduce lung neutrophil load during the acute and chronic phase of the infection (63). This is the first report highlighting neutrophil recruitment *via* the heterocomplex, and future studies are needed to validate its activity in other pathological conditions driven by this cell type.

The research on inhibitors of the CXCL12/HMGB1 heterocomplex was boosted by the results obtained in patients with RA and *in vivo* models of inflammation, in view of developing additional treatments for several pathological conditions fueled by monocyte infiltration (63–66). Currently, small molecules and computationally identified peptides have been tested for their ability to inhibit the heterocomplex *in vitro* and *in vivo.*

### CXCL12/HMGB1 heterocomplex in tissue repair

Two independent works have demonstrated that the CXCL12/HMGB1 heterocomplex enhances tissue regeneration *in vivo* after muscle injury or bone fracture, acting *via* CXCR4 (**Figure 2B**) (67, 68). Tirone and colleagues showed that HMGB1 accelerates tissue regeneration by acting on resident stem cells. This process requires the presence of both CXCL12 and CXCR4, indicating a role for the CXCL12/HMGB1 heterocomplex (67).

Systemically administrated exogenous HMGB1 in C57BL6/J mice accelerates hematopoietic recovery after myeloablation induced by chemotherapy (68). On the other hand, genetic deletion of HMGB1 delays fracture healing. The regenerative process is inhibited by adding glycyrrhizin or AMD3100, suggesting that HMGB1 exerted these regenerative effects by forming the heterocomplex with CXCL12 and acting *via* CXCR4. By investigating the cell cycle rate, cell dimensions, ATP concentrations, mitochondrial DNA, and mTORC1 dependence, Lee and colleagues showed that HMGB1 drives hematopoietic stem cells into the dynamic GAlert phase, defined as an intermediate active state between G0 and G1 (68).

Both groups proposed 3S-HMGB1, a synthetic non-oxidizable form of HMGB1 in which the cysteines are replaced by serines, as a potential new pharmacological tool with a broad range of medical uses, including hematopoietic recovery after chemotherapy, healing after injury or elective surgery.

### CXCL12/HMGB1 heterocomplex in cancer

While the multiple activities orchestrated by CXCL12 in the tumor microenvironment have been well documented, as well as the presence of HMGB1, little is known on the relevance of the CXCL12/HMGB1 heterocomplex in mediating tumor growth and metastasis.

CXCL12 promotes cancer development by two main mechanisms: i) directly activating signaling pathways involved in cancer cell growth, metastasis, and angiogenesis, and ii) indirectly promoting metastasis by recruitment of CXCR4^+^ cancer cells to CXCL12-expressing organs (69).

On the other hand, HMGB1 plays paradoxical roles in cancer, depending on its localization. While nuclear HMGB1 acts as a tumor suppressor for its roles in chromosome stability and induction of tumor cell death, high extracellular HMGB1 expression has been associated with poor prognosis in patients with various type of cancers (70). Although tumor cells mainly express reduced-HMGB1, infiltrating leukocytes are responsible for the presence of oxidized-HMGB1 in tumor microenvironment (52).

Whether tumor cell migration, metastasis formation, and immune cell recruitment can be modulated by the heterocomplex is yet to be demonstrated.

## Naturally arising antibodies against chemokines

A further mechanism by which chemokine activities can be modulated is through the development of autoantibodies targeting their binding or triggering sites (**Figure 1A**).

Arise of anti-cytokines antibodies is well documented in pathology, where they prolong the half-life of circulating cytokines, as observed for interleukin (IL)3, IL4, IL6 and IL7 (71, 72). In addition they trigger Fcγ receptors and stimulate pro-inflammatory responses (73) or promote complement mediated cytotoxicity (74). Similar mechanisms of action could be envisioned for anti-chemokine autoantibodies.

Few reports described the role and functions of anti-chemokine autoantibodies. They can promote or prolong chemokine activities, or be beneficial to the host, through dampening excessive inflammation, thanks to their neutralizing properties (**Table 2**) (78, 79).

**Table 2:** Naturally arising antibodies against chemokines.

Anti-chemokine antibodies sustaining pathology			
Disease	Autoantibody specificity	Mode of action	Reference
Acute respiratory distress syndrome	CXCL8 (immune-complexes)	Associated to adverse outcome Chemotactic activity on neutrophil maintained Superoxide release *via* FcγRIIa	(75) (73)
Asthma	CXCL8 (immune-complexes)	Proinflammatory	(76)
Rheumatoid Arthritis	CXCL8 CCL4, CCL19, CCL25, CXCL7, CXCL8, CXCL9	Proinflammatory; Increased in patients with advanced extra-articular and clinical manifestations	(77)
			(78)
Anti-chemokine antibodies preventing adverse outcome			
Disease	Autoantibody specificity	Mode of action	Reference
Type1 Diabetes Mellitus	CCL3	Neutralizing activity; Counteract disease progression	(79)
Atopic Dermatitis	CCL3	Neutralizing activity; Counteract disease progression	(80)
Prostate Cancer	CCL2	Neutralizing activity; Limit tumor growth	(81)
COVID-19	CCL19, CCL22, CXCL17	Unknown; Increased in COVID-19 convalescents	(78)
	CXCL8, CCL25, CXCL5	Unknown; Increased in COVID-19 convalescents with mild disease	
	CCL21, CXCL13, CXCL16	Neutralizing activity (CXCL13, CXCL16); Increased in COVID-19 convalescents without long COVID	

### Anti-chemokine antibodies sustaining pathology

Acute respiratory distress syndrome (ARDS) is a life threatening pulmonary condition characterized by pulmonary infiltrates, hypoxemia and edema. Bronchoalveolar lavage fluid (BAL) of ARDS patients presents elevated levels of autoantibodies complexed with CXCL8 (82). In a study aimed at investigating the relationship between the presence of these complexes and development stages of acute lung injury, Kurdowska and colleagues demonstrated an association between elevated levels of autoantibodies bound to CXCL8 and an adverse outcome (75). CXCL8-autoantibodies complexes, isolated from BAL after acute lung injury, maintain the CXCL8 chemotactic activity on neutrophils and, in addition, can trigger superoxide release by binding to FcγRIIa (73). Elevated levels of both IgG and IgA autoantibodies complexes with CXCL8 are also found in circulation, as well as in BAL of asthmatic patients, confirming their proinflammatory role (76). Anti-CXCL8 autoantibodies were also identified in RA patients, with high levels of IgA anti-CXCL8 present in patients with advanced extra-articular and clinical manifestations (77). The authors hypothesize that prolonged CXCL8 production leads to the arising of anti-CXCL8 antibodies, aiming at limiting the persistent inflammation; however, the presence of such antibodies complexed with CXCL8, instead, promotes Fc mediated activities, contributing to the persistent chronic inflammation. We recently confirmed elevated levels of circulating anti-CXCL8 IgG autoantibodies in RA patients together with other autoantibodies targeting CCL4, CCL19, CCL25, CXCL7 and CXCL9 (78).

### Anti-chemokine antibodies preventing adverse outcome

Type 1 (insulin-dependent) diabetes mellitus (T1DM) is a chronic autoimmune disease in which insulin-secreting cells within Langerhans islets in pancreas are eliminated by the immune system (83). Patients with T1DM develop autoantibodies against several antigens, which include insulin and islet cells, before symptoms onset. Of note, the majority of T1DM patients selectively develop anti-CCL3 antibodies, with neutralizing properties (79). Anti-CCL3 antibodies production might be specific of T1DM, since the authors did not find them in other autoimmune diseases. Interestingly, anti-CCL3 antibodies are also found in 95% of first-degree relatives of T1DM patients. Cameron and colleagues showed that in CCL3-deficient NOD mice about 60% of the pancreas islets display a normal histology. In contrast, in NOD mice expressing CCL3, 40% of pancreatic islets showed sign of moderate to severe insulitis (84). Indeed, when NOD mice were treated with an anti-CCL3 antibody, a reduction in incidence of diabetes was observed (84). Collectively, these data suggest that, these antibodies might counteract disease progression.

Higher level of autoantibodies against CCL3 were shown in atopic dermatitis (AD), a chronic skin inflammation which leads to skin lesions (80). This is in line with previous studies on AD patients in which CCL3 was shown to be produced by PBMCs (85). The presence of these autoantibodies, if proven to neutralize CCL3 activities, might play a role in suppressing disease progression, similarly to T1DM.

In cancer, several studies highlighted the importance of CCL2 in promoting monocyte migration from the bone marrow to the circulation and ultimately to the tumor site, where they elicit immune suppressive activity and stimulate tumor growth (81, 86–89). Prostate cancer patients present high expression of CCL2 in the tumor, and elevated levels of anti-CCL2 neutralizing autoantibodies (81). Importantly, the presence of anti-CCL2 autoantibodies was exclusive in patients bearing malignant, but not benign tumors. Nevertheless, in a mouse model, CCL2 overexpression induced a sustained autoantibody production and significantly limited tumor growth, suggesting a potential beneficial role of these autoantibodies (81). The authors speculated that a therapeutic strategy aiming at enhancing the pre-existing anti-CCL2 antibodies might be beneficial as the sera concentration of these antibodies might not be elevated enough to completely suppress monocyte infiltration and tumor cell migration.

A novel coronavirus, severe acute respiratory syndrome coronavirus 2 (SARS-CoV-2), emerged in December 2019, causing pneumonia outbreaks worldwide (90). Autoantibodies targeting cytokines and several immune factors have been described in patients with coronavirus disease 2019 (COVID-19) (91). In particular, autoantibodies targeting type I IFN have been found in ~ 10% of life-threatening pneumonia and in ~ 20% of deaths due to COVID-19. We have recently demonstrated that autoantibodies targeting several chemokines, including CCL19, CCL22 and CXCL17, are developed post-SARS-CoV-2 infection. Convalescent individuals who experienced mild COVID-19 have higher levels of autoantibodies targeting CXCL8, CCL25 and CXCL5 compared to those that required hospitalization. We showed that these autoantibodies have neutralizing properties, and therefore could be beneficial in dampening the inflammatory response, preventing hyper-activation of the immune system and tissue damage associated to severe COVID-19. Long-term sequelae (long COVID), spanning from neurological, to respiratory and systemic/inflammatory symptoms, have been reported after SARS-CoV-2 infection (92). We found that autoantibodies to CCL21, CXCL13 and CXCL16 are present in individuals that do not develop long COVID (78). All together these data point to a beneficial role of anti-chemokine antibodies in COVID-19, with their presence correlating with better outcome and lack of long COVID.

## Conclusions

Even though, in modern pharmacology, the γ subfamily of GPCR represents the most successful target of small molecule inhibitors for treating diseases affecting different systems, inhibitors of chemokines and chemokine receptors were unsuccessful for the treatment of inflammatory diseases, where the involvement of the chemokine system plays a key role. The extensive characterization of chemokine expression in tissues, both in physiological and pathological conditions, fostered researchers to look at the chemokine system from a distinct perspective, leading to the identification of additional functions. The complexity of the tissue microenvironment influences chemokine receptor mediated cell recruitment and activities. Natural chemokine antagonism modulates cell recruitment in response to different chemokine gradients. Chemokines can exert a repulsive effect either at different chemokine concentrations or acting on different receptors, contributing to tumor immune evasion or tissue egress. Chemokines and inflammatory molecules, concomitantly expressed, can also synergistically act on cell migration and activities. The presence of autoantibodies targeting chemokines, either neutralizing their activities or promoting Fc-mediated functions, sustain pathology or prevent adverse outcome. Therefore, we believe that all these features should be taken into account for future drug development studies and novel approaches into personalized medicine.

## Author contributions

All authors listed, have made substantial, direct and intellectual contribution to the work, and approved it for publication.
